# Integrative taxonomy of a new species of planarian from the Lake Ohrid basin, including an analysis of biogeographical patterns in freshwater triclads from the Ohrid region (Platyhelminthes, Tricladida, Dugesiidae)

**DOI:** 10.3897/zookeys.313.5363

**Published:** 2013-06-28

**Authors:** Giacinta Angela Stocchino, Ronald Sluys, Paolo Deri, Renata Manconi

**Affiliations:** 1 Department of Science for Nature and Environmental Resources, Via Muroni 25, I-07100, University of Sassari, Sassari, Italy; 2 Naturalis Biodiversity Center, P.O. Box 9514, 2300 RA Leiden, The Netherlands; 3 Department of Biology, S.S. 12 Abetone e Brennero 4, I- 56127, University of Pisa, Pisa, Italy

**Keywords:** Platyhelminthes, Tricladida, *Dugesia*, integrative taxonomy, ancient lake, Ohrid, new species, endemicity

## Abstract

A new species of the genus *Dugesia* is described from the Lake Ohrid region in the western part of the Balkan Peninsula, forming the first fully documented species description for this genus in the Ohrid area. The morphological species delimitation is supported by complementary molecular, karyological, and cytogenetic data available from the literature. Therefore, species delineation is based on a truly integrative approach. Further, a short account on the degree of freshwater planarian endemicity in the Ohrid region is provided.

## Introduction

The oligotrophic karstic Lake Ohrid is located in the western part of the Balkan Peninsula on the Macedonian-Albanian frontier. With a limnological age of 2-5 million years it is considered to be one of the oldest lakes in Europe (Albrecht and Wilke 2008). The lake is characterized by a high degree of biodiversity and endemicity in several groups of organisms (Stanković 1960, Albrecht and Wilke 2008). With more than 210 known endemic species it is probably the most biodiverse lake in the world, at least when one takes surface area into account (Albrecht and Wilke 2008). Due to its peculiarities, Lake Ohrid is considered to be a key site for biodiversity and speciation research (Albrecht and Wilke 2008).

The first studies on the triclad fauna of the Ohrid area date back to the 1920’s with the first description of several new species of *Phagocata* Leidy, 1847 and *Dendrocoelum* Örsted, 1844 (cf. Stanković and Komárek 1927). Further important researches carried out during the 20th century, mainly by Stanković (1938, 1960, 1969) and Kenk (1978), contributed to a better knowledge of this very interesting planarian fauna. In his valuable monograph, Stanković (1960) in particular pointed out the extraordinary biogeographical situation of the endemic triclads in the Ohrid region.

In this paper we report on a new species of freshwater planarian of the genus *Dugesia*, forming the first fully documented species description for this genus in the Ohrid area. Our morphological species delimitation was supported by complementary molecular, karyological, and cytogenetic data available from the literature. Therefore, our species delineation is based on a truly integrative approach. Further, we provide a short account on the biogeographical patterns in freshwater planarians and their degree of endemicity in the Ohrid region.

## Materials and methods

**Figure 1.**
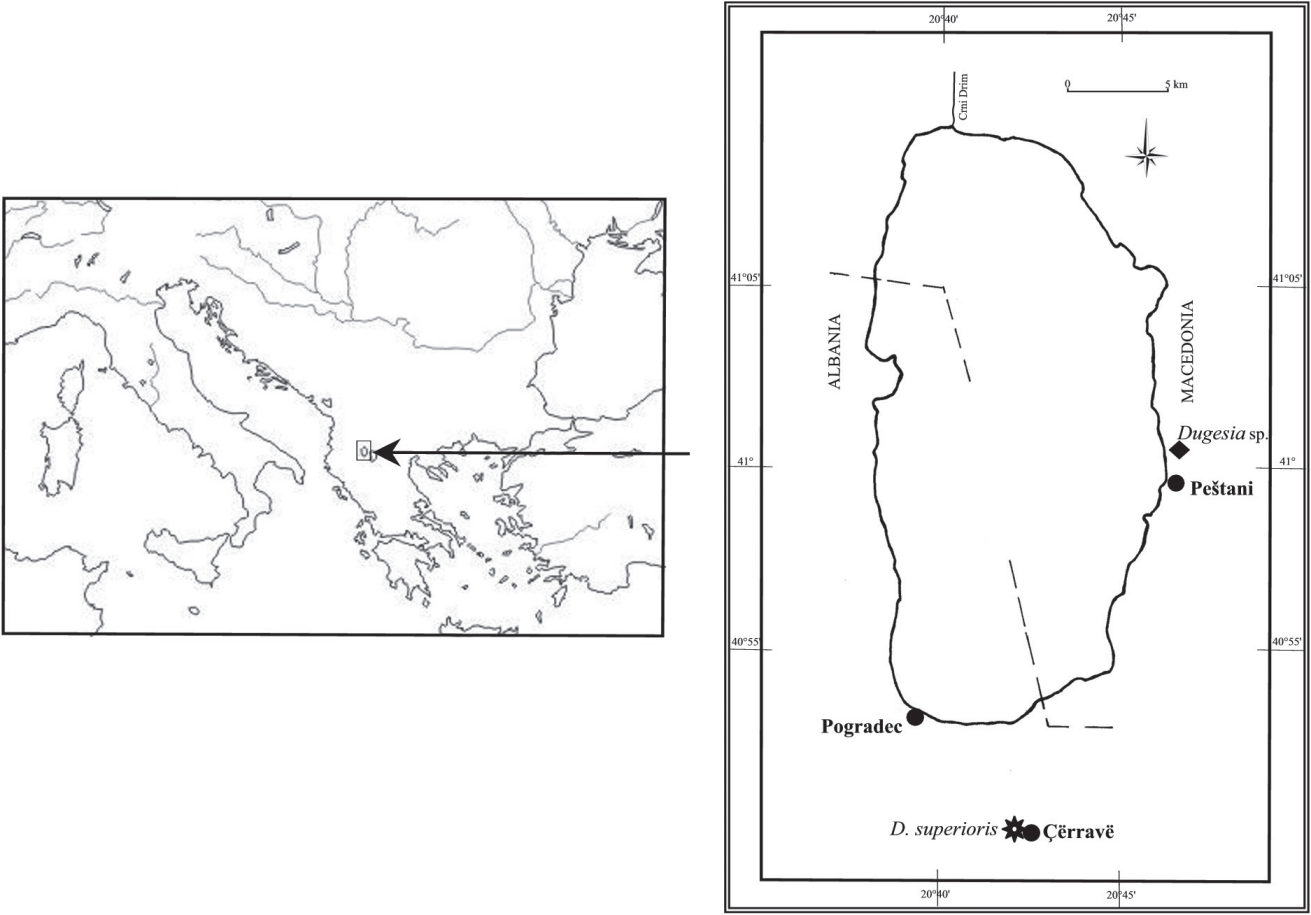
Geographic distribution of *Dugesia superioris* (indicated by an asterisk) and *Dugesia* sp. NMNH 55294 (indicated by black diamond) in the Lake Ohrid region.

Planarians were collected in 1995 from the southern section of Lake Ohrid basin, near the town of Çërravë, along the Pogradec-Korçë road, ca. 10 km south-east of Pogradec, at an altitude of ca. 800 m asl (Fig. 1). The animals were found under pebbles and among vegetation in a rivulet, flowing along a steep meadow, joining a tributary stream of the lake. All individuals (n = 20) were asexual at collection. The collected specimens were transferred to the laboratory, reared in glass bowls under semi-dark conditions at 18 +/- 2 °C and fed with fresh beef liver.

After having been kept in the laboratory for about one year, during which the strain notably increased in numbers due to asexual reproduction by fission, approximately 30% of the specimens shifted from the fissiparous reproductive mode towards a tendency to sexualize, i.e. to develop reproductive organs. These sexualized animals displayed the characteristic features of ex-fissiparous individuals: large body size, deve-lopment of the copulatory apparatus, hyperplasic ovaries.

For morphological study sexualized specimens were fixed for 24 hours in Bouin’s fluid, dehydrated in a graded ethanol series, cleared in toluene, and embedded in synthetic paraffin. Serial sections were made at intervals of 5–7 μm and were stained with Harris’ haematoxylin-eosin, Mallory’s trichrome, or Pasini’s reagent.

The material is deposited in the Naturalis Biodiversity Center, Leiden, The Netherlands (collection code: ZMA), and in the Giacinta A. Stocchino collection (CGAS), University of Sassari.

### Abbreviations used in the figures

bc: bursal canal; bg: bulb glands; ca: common atrium; cb: copulatory bursa; cg: cement glands; cm: circular muscles; d: diaphragm; e: epithelium; ed: ejaculatory duct; g: gonopore; gd: gonoduct; ie: infranucleate epithelium; l: lumen; lm: longitudinal muscles; lod: left oviduct; ma: male atrium; pb: penis bulb; pf: penial fold; pg: penis papilla glands; ph: pharynx; pp: penis papilla; rod: right oviduct; s: spermatophore; sg: shell glands; sv: seminal vesicle; vd: vas deferens.

## Results

### Systematic Account Order Tricladida Lang, 1884 Suborder Continenticola Carranza, Littlewood, Clough, Ruiz-Trillo, Baguñà & Riutort, 1998 Family Dugesiidae Ball, 1974 Genus *Dugesia* Girard, 1850

#### Dugesia superioris

Stocchino & Sluys sp. n.

urn:lsid:zoobank.org:act:E1A595E2-6466-4F59-99CF-3E846A545332

http://species-id.net/wiki/Dugesia_superioris

Figs 1 [Fig F2] [Fig F3] –4 ; Table 1

##### Material examined.

**Holotype**: ZMA V.Pl. 7153.1, Çërravë, Pogradec District (40°50'56"N, 20°42'60"E), Lake Ohrid basin, Albania, August 1995, coll. P. Deri and N. Mazniku, one set of sagittal sections on 50 slides (stained in Harris’ haematoxylin-eosin).

**Paratypes**: CGAS Pla 6. 1, ibid., sagittal sections on 43 slides (stained in Harris’ haematoxylin-eosin); CGAS Pla 6. 2, ibid., sagittal sections on 12 slides (stained in Mallory’s trichrome); CGAS Pla 6. 3 ibid., transverse sections on 135 slides (stained in Pasini’s reagent); ZMA V.Pl. 7153.2, ibid., transverse sections on 131 slides (stained in Harris’ haematoxylin-eosin); CGAS Pla 6. 4, ibid., transverse sections on 60 slides (stained in Harris’ haematoxylin-eosin); ZMA V.Pl. 7153.3, ibid., horizontal sections on 21 slides (stained in Harris’ haematoxylin-eosin).

##### Diagnosis.

*Dugesia superioris* is characterized by the presence of the following features: dorsal course of the ejaculatory duct; subterminal opening of the ejaculatory duct; asymmetrical openings of the oviducts into the bursal canal; openings of vasa deferentia at halfway along the seminal vesicle; plump penis papilla; small diaphragm; triploid chromosome complement of 24 + 1B-chromosomes.

##### Description.

**Figure 2. F2:**
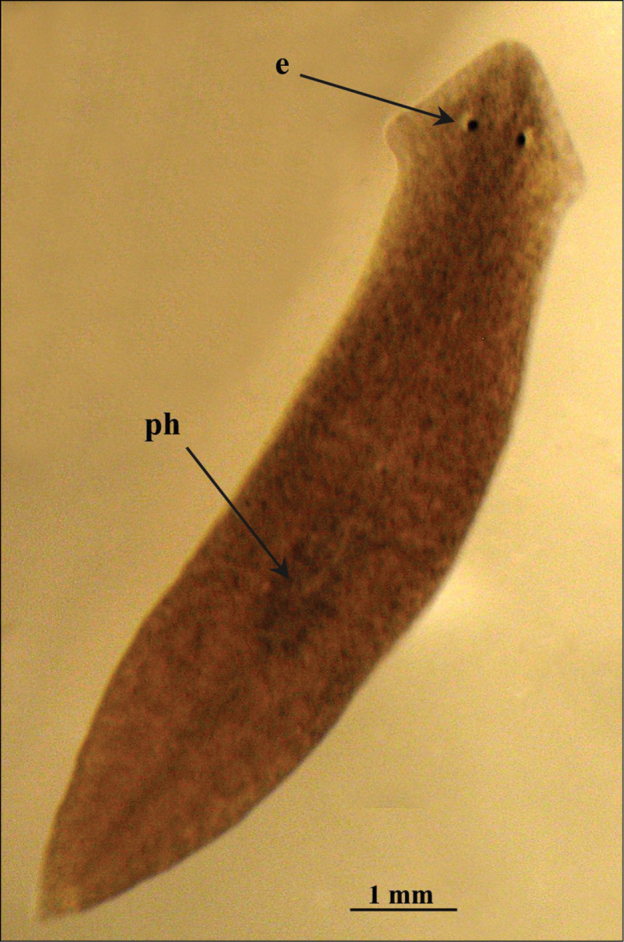
*Dugesia superioris*. Habitus of a living fissiparous specimen.

Body size of living fissiparous specimens ranged from 7–10 mm in length and 1.5–2 mm in width (Fig. 2). Sexualized specimens were about 13–16 mm in length and about 3 mm in width. Two eyes are present in the middle of the head, and unpigmented auricular grooves are marginally placed just posteriorly to the eyes. The colour is uniformly brown dorsally, and pale ventrally.

Inner and outer pharyngeal musculature is bilayered, i.e. without an extra, third, outer longitudinal muscle layer. The ovaries are hyperplasic, with several scattered masses at a short distance behind the brain, filling up the entire dorso-ventral space. A degenerative condition is clearly evident in the ovaries, in that maturation of the oocytes is regular up to the beginning of the diplotene stage, whereas diplotenic oocytes show progressive cytoplasm vacuolation, followed by collapse of the entire cell content and by cell necrosis.

**Figure 3. F3:**
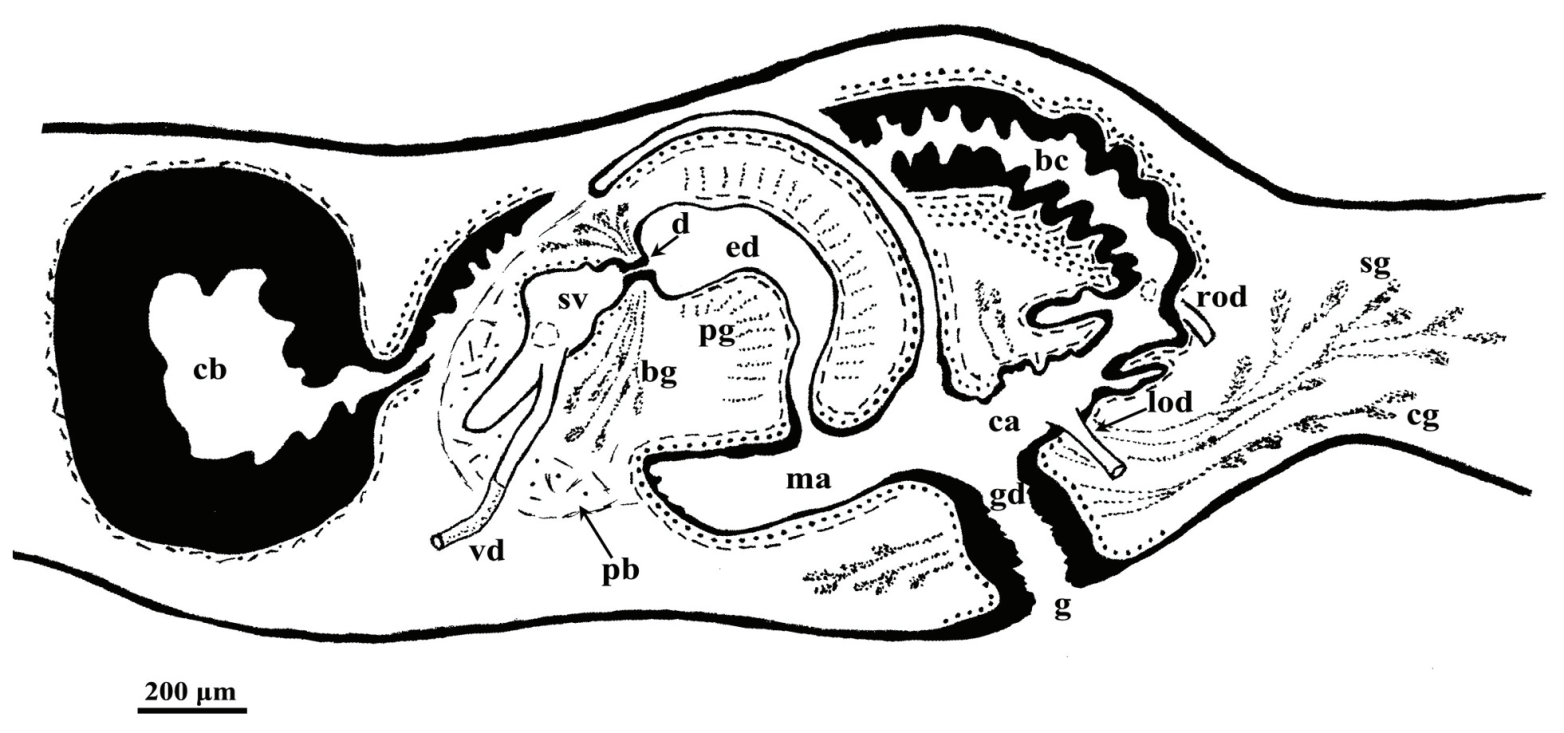
*Dugesia superioris*. Holotype ZMA V.Pl. 7153.1, sagittal reconstruction of the copulatory apparatus (anterior to the left).

The anterior portion of the infranucleated oviducts is expanded to form a seminal receptacle that arises in the middle of the ovarian masses at a poorly defined position, dependent upon the hyperplasic condition of the ovaries. The oviducts run ventrally in a caudal direction up to the vaginal area and open asymmetrically into the distal section of the bursal canal. The right oviduct opens dorsally to the left one. The latter opens very close to the point where the canal communicates with the common atrium (Fig. 3). The very abundant shell glands open at the level of the left oviducal opening.

The testes are situateddorsally and extend from just anterior to the ovaries to the posterior end of the body. The testes generally are under-developed in that the majority of germ cells are represented only by spermatogonia (ca. 90%). In only some specimens, and then in only a few follicles, mature sperms are present. However, in all cases anomalies were observed, such as irregularly shaped spermatids and spermatozoa. Vitellaria are located between the testes and the intestinal branches.

**Figure 4.**
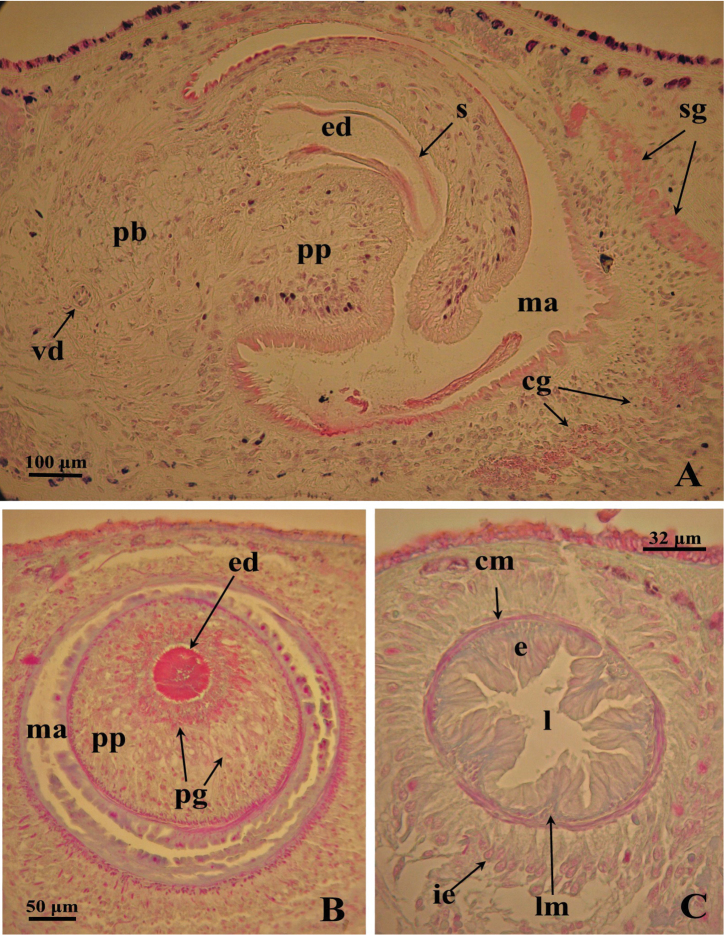
*Dugesia superioris*. Photomicrographs of the copulatory apparatus. **A** Holotype ZMA V.Pl. 7153.1, sagittal section showing the penis bulb and the penis papilla with the ejaculatory duct **B** Paratype CGAS Pla 6. 3, transverse section of the penis papilla and the ejaculatory duct surrounded by numerous glands **C** Paratype CGAS Pla 6. 3, transverse section of the bursal canal.

The large sac-shaped copulatory bursa is lined by a columnar, glandular epithelium bearing basal nuclei and it is surrounded by a thin layer of muscles. From the mid-posterior wall of the bursa the bursal canal runs in a caudal direction, to the left of the copulatory apparatus. Posteriorly to the gonopore the bursal canal recurves antero-ventrally and, subsequently, opens into the posterior section of the atrium. The bursal canal is lined by a pleated epithelium with cylindrical, infranucleated, and ciliated cells and is surrounded by a thin, subepithelial layer of longitudinal muscles, followed by a thicker layer of circular muscle. Ectal reinforcement is absent (Figs 3, 4C). At its distal section, near the atrium, the bursal canal shows several deep folds.

The moderately developed penis bulb, rich in glands, consists of intermingled longitudinal and circular muscle fibres. It houses an elongated seminal vesicle, which extends through the entire length of the penis bulb. The anterior half of the seminal vesicle is tubular in shape, while its distal, posterior section is considerably expanded.

The vasa deferentia penetrate the antero-lateral wall of the penis bulb and open separately and symmetrically into the seminal vesicle at a position about halfway along the vesicle. No spermiducal vesicles were observed in any of the specimens examined. The seminal vesicle, lined with a flat epithelium and surrounded in its distal, posterior section by layers of circular muscle fibres, opens into the ejaculatory duct via a small diaphragm. The latter, located at the base of the penis papilla, receives the openings of very abundant bulb glands. The blunt penis papilla is lined with an infranucleated epithelium that is underlain with a thin subepithelial layer of circular muscles fibres, followed by a layer of longitudinal muscle fibres.

The ejaculatory duct follows a dorsally displaced course through the penis papilla and has a sub-terminal opening. The spacious lumen of the ejaculatory duct is lined by a cuboidal, infranucleated epithelium that is surrounded by a layer of longitudinal musclesand receives the abundant secretion of penis papillaglands; in the majority of examined specimens the ejaculatory duct contained an empty spermatophore (Fig. 4A, B). Both the bulb glands and the penis papilla glands secrete globules that stain purple in Pasini’s reagent (Fig. 4B). The acentral, dorsally displaced ejaculatory duct makes the penis papilla asymmetrical, with the ventral part being thicker than the dorsal one (Figs 3, 4A, B).

The genital atrium is lined by an infranucleated epithelium that is underlain by a subepithelial layer of circular muscle, followed by a layer of longitudinal muscle fibres. The common atrium communicates with a gonoduct that is lined by a columnar epithelium, which receives the openings of very abundant cement glands; the gonoduct communicates with the ventral gonopore (Fig. 3).

##### Etymology.

The specific epithet is derived from the Latin *superius*, located at a higher position, and alludes to the dorsally displaced course of the ejaculatory duct in the penis papilla.

##### Geographical distribution.

Known from the type locality and, most likely, also from a second Albanian locality, viz. Voskopojë (see below).

**Table 1.** Checklist of Tricladida from the Lake Ohrid hydrographic basin.<br/>

**Taxa**	**Lacustrine habitat**	**Adjacent waters of lake Ohrid**	**Endemic species**	**References**
**Dugesiidae Ball, 1974**				
***Dugesia* Girard, 1850**				
*Dugesia superioris*	**_**	tributary rivulet	–	Present paper
*Dugesia* sp.	**_**	Spring Elešec	?	Kenk 1978
*Dugesia gonocephala* (Dugès, 1830)(?)	**_**	tributary streams and the effluent Crni Drim River	**_**	Stanković 1960
***Schmidtea* Ball, 1974**				
*Schmidtea lugubris* (Schmidt, 1861)	**_**	stagnant waters of the Ohrid region; drainage ditch at Teferić	**_**	Stanković 1960; Kenk 1978
**Dendrocoelidae Hallez, 1892**				
***Dendrocoelum* Örsted, 1844**				
*Dendrocoelum adenodactylosum* (Stanković & Komárek, 1927)	littoral, sublittoral, profundal zones	littoral cold springs; tributary streams; a tributary of the effluent Crni Drim River	**_**	Stanković and Komárek 1927; Kenk 1978
*Dendrocoelum albidum* Kenk, 1978	sublittoral zone	**_**	**+**	Kenk 1978
*Dendrocoelum cruciferum* (Stanković, 1969)	sublittoral zone	**_**	**+**	Stanković 1969; Kenk 1978
*Dendrocoelum decoratum* Kenk, 1978	sublittoral and profundal zones	**_**	**+**	Kenk 1978
*Dendrocoelum dorsivittatum* Kenk, 1978	profundal zone	**_**	**+**	Kenk 1978
*Dendrocoelum jablanicense* (Stanković & Komárek, 1927)	**_**	Šum Spring; tributary streams	**+**	Stanković and Komárek 1927; Stanković 1960; Kenk 1978
*Dendrocoelum komareki* (Stanković, 1969)	sublittoral zone	**_**	**+**	Stanković 1969; Kenk 1978
*Dendrocoelum lacteum* (Müller, 1774)	sublittoral and profundal zones	stagnant waters	**_**	Stanković and Komárek 1927; Arndt 1938; Stanković 1960; Kenk 1978
*Dendrocoelum lacustre* (Stanković, 1938)	sublittoral zone	**_**	**+**	Stanković 1938; Stanković 1969; Kenk 1978
*Dendrocoelum lychnidicum* (Stanković, 1969)	sublittoral zone	**_**	**+**	Stanković 1969; Kenk 1978
*Dendrocoelum maculatum* (Stanković & Komárek, 1927)	littoral zone	tributary streams; littoral springs	**+**	Stanković 1960; Kenk 1978
*Dendrocoelum magnum* (Stanković, 1969)	sublittoral zone	**_**	**+**	Stanković 1969; Kenk 1978
*Dendrocoelum minimum* Kenk, 1978	profundal zone	**_**	**+**	Kenk 1978
*Dendrocoelum ochridense* (Stanković & Komárek, 1927)	littoral, sublittoral, profundal zones	**_**	**+**	Stanković 1960; Kenk 1978
*Dendrocoelum sanctinaumi* (Stanković & Komárek, 1927)	littoral and sublittoral zones	tributary streams; littoral springs	**+**	Stanković 1960; Kenk 1978
*Dendrocoelum sinisai* Kenk, 1978	profundal zone	**_**	**+**	Kenk 1978
*Dendrocoelum translucidum* Kenk, 1978	profundal zone	**_**	**+**	Kenk 1978
**Planariidae Stimpson, 1857**				
***Crenobia* Kenk, 1930**				
*Crenobia alpina montenegrina* (Mrázek, 1904)	**_**	springs; tributary streams and the effluent Crni Drim River	_	Stanković and Komárek 1927; Stanković 1960; Kenk 1978
***Phagocata* Leidy, 1847**				
*Phagocata maculata* (Stanković, 1938)	sublittoral zone	_	**+**	Stanković 1938; Stanković 1960; Kenk 1978
*Phagocata ochridana* (Stanković & Komárek, 1927)	littoral, sublittoral, profundal zones	springs and pools	**+**	Stanković and Komárek 1927; Stanković 1960; Kenk 1978
*Phagocata stankovici* (Reisinger, 1960)	sublittoral and profundal zones	_	**+**	Reisinger 1960; Kenk 1978
*Phagocata undulata* (Stanković, 1960)	sublittoral zone	_	**+**	Stanković 1960; Kenk 1978
***Planaria* Müller, 1776**				
*Planaria torva* (Müller, 1774)	**_**	only one specimen at the mouth of the Studenčišta brook	**_**	Stanković 1960; Kenk 1978
***Polycelis* Ehrenberg, 1831**				
*Polycelis tenuis* Ijima, 1884	**_**	tributary streams	_	Stanković 1960; Kenk 1978

### Additional data supporting the status of the new species

A karyological study by Deri et al. (1999) identified for the Pogradec population a complement of 24 standard chromosomes with one B-chromosome, suggesting a tri-ploid condition with a haploid number of n = 8. Moreover, their karyometric analysis indicated a probably aneutriploid condition, due to a constant excess of small, medium-sized chromosomes. A haploid number with n = 8 represents the most common chromosome number among *Dugesia* species. *Dugesia superioris* shares the tri- ploid condition with a haploid number of n = 8 with only a few other species from the Western Palaearctic Region, viz. *Dugesia benazzii* Lepori, 1951, *Dugesia etrusca* Benazzi, 1946, *Dugesia liguriensis* De Vries, 1988a, and *Dugesia subtentaculata* (cf. Benazzi and Benazzi-Lentati 1976, Ribas 1990, Pala 1993, cf. Lázaro et al. 2009).

A molecular cytogenetic comparison of several species and populations of the genus *Dugesia* revealed that these planarians from Pogradec besides two telomeric NOR *loci*, also have a ribosomal site located in an intercalated position on the long arm of one of the largest chromosomes (Batistoni et al. 1999). This peculiar condition differs from other planarian taxa, in which 18S + 28S rRNA genes appeared preferentially located on telomeric regions of medium-sized chromosomes, and was interpreted by the authors as a structural chromosomal rearrangement, such as a paracentric inversion, suggesting a case of speciation.

More recently, a phylogeographic analysis of two Albanian populations, one from Pogradec and the other from Voskopojë (populations 30 and 31, respectively in Lázaro et al. 2009), revealed that they belong to the same clade, which is well-separated from other species and populations of *Dugesia* in the Western Mediterranean region, thus pointing to a new species (Lázaro et al. 2009). In a second study, which included other and more eastern Mediterranean species of *Dugesia*, the population from Pogradec (population 15 in Solà et al. 2013) also sat on its own branch, separate from all other populations of *Dugesia* examined.

### Previous records of *Dugesia* from the Lake Ohrid region

**Figure 5.**
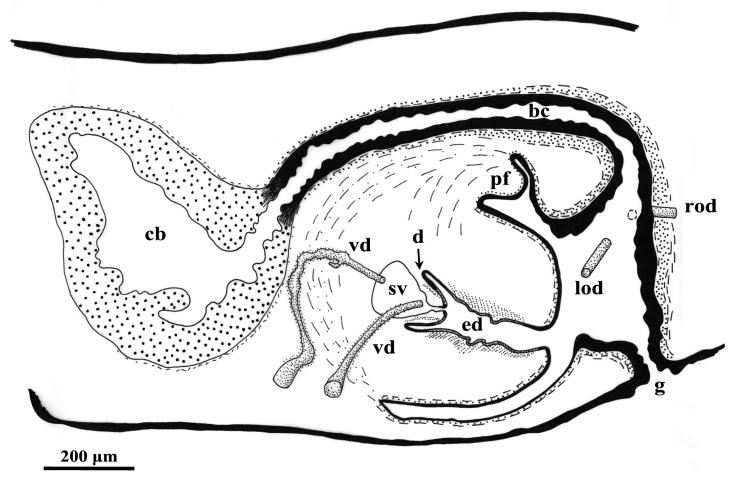
Sagittal reconstruction of the copulatory apparatus of *Dugesia* specimen NMNH 55294.

From Lake Ohrid only one species of *Dugesia* has been reported until now, viz. *Dugesia gonocephala*. Stanković (1960) reported the species from running waters and springs in the Ohrid region,but he did not mention exact localities. However, in view of the fact that in that period all continental European planarians with a triangular head were assigned to this species, the taxonomic status of these Ohrid planarians remains uncertain. Kenk (1978) reported the presence of *Dugesia gonocephala* in the Spring Elešec, about 2 km north of Peštani (Fig. 1). Re-examination of his material (NMNH 55294) revealed that the male copulatory apparatus of the animal consists of a stubby penis papilla, provided with a small, dorsal penial fold (Fig. 5). The diaphragm is rather large and pointed and projects into a broad, central ejaculatory duct, which opens at the tip of the penis papilla. The vasa deferentia open into an intrapenial seminal vesicle, albeit that the precise location of the openings is different. One vas deferens opens close to the diaphragm, whereas the other duct opens through the anterior lining epithelium of the seminal vesicle. A large copulatory bursa is situated immediately behind the pharyngeal pocket. The bursal canal is lined with a tall, nucleated epithelium and is surrounded by a subepithelial layer of longitudinal muscles, followed by a layer of circular muscle. In the vaginal area and on the posterior, curved section of the bursal canal the circular muscle layer is well-developed but becomes gradually thinner on the remaining part of the canal. Ectal reinforcement in the form of an extra, outer layer of longitudinal muscle is present in the vaginal region of the bursal canal. The oviducts open into the most proximal section of the bursal canal, one oviducal opening at the point where the canal communicates with the atrium, while the opening of the other oviduct is located somewhat more dorsally. Shell glands could not be discerned.

### Biogeographical patterns of freshwater triclads in the Ohrid region

Out of a total of 27 nominal species of triclads reported from the Lake Ohrid region, 19 are endemic to this area (70% of endemicity) (Table 1). Most of these endemics (15) are restricted to the lake proper: *Dendrocoelum albidum* Kenk, 1978, *Dendrocoelum cruciferum* (Stanković, 1969), *Dendrocoelum decoratum* Kenk, 1978, *Dendrocoelum dorsivittatum* Kenk, 1978, *Dendrocoelum komareki* (Stanković, 1969), *Dendrocoelum lacustre* (Stanković, 1938), *Dendrocoelum lychnidicum* (Stanković, 1969), *Dendrocoelum magnum* (Stanković, 1969), *Dendrocoelum minimum* Kenk, 1978, *Dendrocoelum ochridense* (Stanković & Komárek, 1927), *Dendrocoelum sinisai* Kenk, 1978, *Dendrocoelum translucidum* Kenk, 1978, *Phagocata maculata* (Stanković, 1938), *Phagocata stankovici* (Reisinger, 1960), *Phagocata undulata* (Stanković, 1960). The species that live in the lake may inhabit only one of the three major bathymetrical zones of the lacustrine bottom (littoral, sublittoral and profundal) or can be found in two or more zones (Table 1). Onlythreespecies are endemic bothto the lake and adjacent water systems: *Dendrocoelum maculatum* (Stanković & Komárek, 1927), *Dendrocoelum sanctinaumi* (Stanković & Komárek, 1927), *Phagocata ochridana* (Stanković and Komárek, 1927).

*Dendrocoelum lacteum* (Müller, 1774) is a species with a very large distributional range across the Palaearctic Region that occursboth in the lake and in surrounding waters. Sywula et al. (2006) showed that *Dendrocoelum lacteum* from Lake Ohrid is genetically distant from the Central European populations and suggested that the Ohrid population should be considered as a distinct species. However, this study was based only on allozyme data, while its results do not fully support the conclusion of the authors. For example, the genetic distance between the Ohrid population of *Dendrocoelum lacteum* and *Dendrocoelum adenodactylosum* (Stanković & Komárek, 1927) is of the same order of magnitude as the distance to the Central European populations, whereas in the phylogenetic trees the Ohrid population strongly clusters with *Dendrocoelum adenodactylosum*.

*Dendrocoelum adenodactylosum* is very common in the lake, in its tributary streams and springs and also in Lake Prespa, a nearby lake southeast of Lake Ohrid that is a major water supplier for the latter. Six species are found in surrounding streams and springs and do not occur in the lake proper, viz. *Dugesia superioris*, *Dendrocoelum jablanicense* (Stanković & Komárek, 1927), *Schmidtea lugubris* (Schmidt, 1861), *Crenobia alpina montenigrina* (Mrázek, 1904), *Planaria torva* (Müller, 1774), and *Polycelis tenuis* Ijima, 1884. *Dendrocoelum jablanicense* is endemic of the Lake Ohrid region, while the others concern widespread species.

## Discussion

*Dugesia superioris* differs from its congeners in particular in (a) the dorsal course of the ejaculatory duct, with its sub-terminal opening, (b) the asymmetrical openings of the oviducts into the bursal canal, and (c) the openings of vasa deferentia at about halfway along the seminal vesicle.

For the genus *Dugesia* a dorsal course of the ejaculatory duct was reported for the first time by Stocchino et al. (2005) for the endemic Sardinian species *Dugesia hepta* Pala, Casu & Vacca, 1981. However, in this species the opening of the duct is located laterally on the right side, near the tip of the penis papilla. Moreover, this species is characteri-zed by a ventro-lateral penial fold, which is absent in the new species. *Dugesia superioris* therefore represents the second species of the genus showing a dorsal course of the ejaculatory duct. Further, another important difference between *Dugesia hepta* and *Dugesia superioris* is the haploid chromosome number, which counts n = 7 in the former (Pala et al. 1981) and n = 8 in the latter (Deri et al. 1999, see below).

A subterminal opening of the ejaculatory duct, as found in *Dugesia superioris*, occurs in no less than 26 species of *Dugesia*: *Dugesia bakurianica* Porfirjeva, 1958, *Dugesia biblica* Benazzi & Banchetti, 1972, *Dugesia leporii* Pala et al., 2000, and *Dugesia sicula* Lepori, 1948, from the Western Palaearctic; *Dugesia aethiopica* Stocchino et al., 2002, *Dugesia arabica* Harrath & Sluys, 2013, *Dugesia astrocheta* Marcus, 1953, *Dugesia lanzai* Banchetti & Del Papa, 1971, *Dugesia lamottei* De Beauchamp, 1952, *Dugesia neumanni* (Neppi, 1904) and *Dugesia myopa* De Vries, 1988b from the Afrotropical Region; the other 15 species are distributed in the Oriental Region, Eastern Palaearctic and Australasian Region, viz. *Dugesia andamanensis* (Kaburaki, 1925), *Dugesia austroasiatica* Kawakatsu, 1985, *Dugesia batuensis* Ball, 1970, *Dugesia bengalensis* Kawakatsu, 1983, *Dugesia burmanensis* (Kaburaki, 1918), *Dugesia deharvengi* Kawakatsu & Mitchell, 1989, *Dugesia indica* Kawakatsu, 1969, *Dugesia indonesiana* Kawakatsu, 1973, *Dugesia japonica* Ichikawa & Kawakatsu, 1964, *Dugesia leclerci* Kawakatsu & Mitchell, 1995, *Dugesia lindbergi* De Beauchamp, 1959, *Dugesia nannophallus* Ball, 1970, *Dugesia novaguineana* Kawakatsu, 1976, *Dugesia tamilensis* Kawakatsu, 1980, and *Dugesia uenorum* Kawakatsu & Mitchell, 1995. However, in all of these species the ejaculatory duct is ventrally displaced, except for *Dugesia bakurianica* in which the ejaculatory duct is central. Therefore, a dorsal course of the ejaculatory duct and a subterminal opening of the duct represents a new diagnostic combination in the genus *Dugesia*.

The Pogradec population had already been subjected to karyological, cytogenetic, and phylogeographic studies before anything was known about the anatomy of the specimens (see above). All of these analyses pointed to a situation that this *Dugesia* population differs considerably from congeneric populations. Therefore, it was unsurprising that the anatomy of the Pogradec animals suggested also that they represent a new species. As a result of the cumulation of the evidences from these independent datasets, the present delineation of the new species is based on a truly integrative approach to taxonomy.

Studies on the phylogeny of *Dugesia* (Sluys et al. 1998 and references therein) considered the asymmetrical penial papilla to constitute an important taxonomic feature. However, this asymmetry related to the apomorphic presence of a ventral ejaculatory duct. Our present study shows that in future analyses this asymmetry needs to be specified by adding a third character state to character (1) (Sluys et al. 1998, p. 277 and Table II), i.e. ejaculatory duct located dorsally.

An asexual population of *Dugesia* sp. was collected in 2006 by R. Manconi from Voskopojë, an Albanian locality situated south-west of Lake Ohrid. Unfortunately, we have been unable to ascertain the taxonomic status of this population due to the lack of sexual specimens (Stocchino and Manconi, pers. obs.). However, according to the phylogeographic analysis of Lázaro et al. (2009) this population is molecularly identical to the Pogradec population and therefore should be assigned also to *Dugesia superioris*. It is noteworthy that the Voskopojë locality is outside of the Ohrid basin and therefore signals a wider distribution of *Dugesia superioris*.

That Kenk (1978) identified his *Dugesia* material from Ohrid (NMNH 55294) as *Dugesia gonocephala* is hardly surprising in view of the fact that at that time many European populations were assigned to *Dugesia gonocephala* *sensu lato.* The precise anatomy of *Dugesia gonocephala* *sensu stricto* was only resolved by De Vries and Ball (1980) and De Vries (1984a, 1986). A comparison with Kenk’s specimen quickly learns that this animal does not conform to *Dugesia gonocephala* because it does not exhibit the muscular ridges, the elongated penis papilla, or the two penial folds (cf. De Vries and Ball 1980, De Vries 1984a). In the presence of a small dorsal penial fold and a central ejaculatory duct the animal resembles *Dugesia benazzii* Lepori, 1951, *Dugesia elegans* De Vries, 1984, *Dugesia taurocaucasica* (Livanov, 1951) and *Dugesia effusa*, the latter recently described from the Greek island Chios (Sluys et al. in prep.). *Dugesia benazzii* from Corsica and Sardinia is characterized by a pointed diaphragm and a penial fold, the position of which is variable but which is usually located dorsally; the size of the penial fold is also variable (Lepori 1951, De Vries 1984b). In *Dugesia benazzii* ectal reinforcement is restricted to the region of the oviducal openings, the latter being symmetrically arranged. In contrast, in the NMNH 55294 specimen the oviducts open asymmetricaly into the bursal canal, while the ectal reinforcement extends further on the bursal canal.

*Dugesia elegans* from Rhodes differs from NMNH 55294 in the presence of a much larger seminal vesicle, a stubbier diaphragm, and the situation that its bursal canal epithelium is infranucleated (De Vries 1984a).

The penial fold of *Dugesia taurocaucasica* is considerably larger than the one in NMNH 55294, while the fold is also traversed by the abundant secretion of cyanophilic glands, which discharge through the lining epithelium of the penial fold. Furthermore, in *Dugesia taurocaucasica* the ectal reinforcement layer on the bursal canal extends for a considerable distance towards the copulatory bursa (Porfirjeva and Dyganova 1987).

The species *Dugesia effusa* differs from NMNH 55294 in the presence of a short, valve-like diaphragm, a large intrabulbar seminal vesicle, a highly glandular penis papilla, and symmetrical oviducal openings into the bursal canal.

Therefore, the *Dugesia* specimen NMNH 55294 may well represent a new species. However, on the basis of only the presently available material we refrain from describing it as new. Furthermore, the asymmetrical openings of the vasa deferentia into the seminal vesicle of this animal represents a highly unusual condition for a species of *Dugesia* and needs to be checked on additional material.

Present data support Stanković’ (1960) suggestion that two faunistic complexes may be distinguished in the Lake Ohrid region, viz. (1) lacustrine endemic forms and (2) inhabitants of other waters outside of the lake with a wider distributional range. In the Ohrid region it is evident that there is a very low degree of exchange between the lacustrine endemic fauna and the non-endemic fauna. Further, the distribution of triclads in the Lake Ohrid area supports the situation for the lake’s fauna in general, namely that endemism occurs at different spatial scales, ranging from species endemic to some parts of the lake to species endemic to the whole Ohrid basin (Albrecht and Wilke 2008).

## Supplementary Material

XML Treatment for Dugesia superioris
